# Waking Up to Monkeypox in the Midst of COVID-19

**DOI:** 10.7759/cureus.30920

**Published:** 2022-10-31

**Authors:** Maleesha Jayasinghe, Dilushini Caldera, Omesh Prathiraja, Abdul Mueez Alam Kayani, Ozair S Siddiqui, James Anwar Coffie-Pierre, Dilka Sewwandi Abeysundara, Avinash Sewsurn, Imesha Hewavitharana, Rahul Jena

**Affiliations:** 1 Medicine, Nanjing Medical University, Nanjing, CHN; 2 Internal Medicine, Nanjing Medical University, Nanjing, CHN; 3 Medicine and Surgery, Nanjing Medical University, Nanjing, CHN; 4 Medicine and Surgery, Allama Iqbal Medical College, Lahore, PAK; 5 Medicine, Gujarat Medical Education and Research Society (GMERS) Medical College and Hospital, Patan, IND; 6 Medicine, Bharati Vidyapeeth Medical College/Bharati Hospital, Pune, IND

**Keywords:** monkeypox virus, outbreak management, health policy making, viral exanthem, corona virus disease, quarantine, world pandemic, vaccination policy, smallpox, hiv diseases

## Abstract

The first incidence of the monkeypox virus (MPXV) was reported in a Danish research facility. Even though first discovered in monkeys, rodents account for the largest reservoir of the disease. It is an encapsulated, brick-shaped double-stranded DNA virus strongly related to the smallpox virus. The risk of acquiring MPXV has been found to be inversely related to smallpox vaccination. Although the cases were initially restricted to African countries, they were first reported outside Africa in the early 2000s. MPXV is transmitted through close personal contact, most commonly through direct skin-skin contact. The fatality rates associated with the MPXV tend to vary in different regions, with Congo clad basin having the highest mortality rate. The majority of the cases of MPXV have been reported in men who have sex with men.

Although optimal infection control and treatment strategies are under investigation, the current management focus is on immunization and the isolation of patients. Effective control strategies are based on implementing a method of contact tracing, quarantining exposed and infected individuals, and using vaccines. There is no proven cure for MPXV, and most infected patients recover without medical intervention. Extensive studies are being conducted to determine the efficacy of antivirals in managing MPXV, with tecovirimat being the first antiviral medication approved by the Food and Drug Administration (FDA) to manage MPXV. The smallpox vaccine has traditionally been thought of as the most effective method of controlling the infection, possibly due to the similarities between the two viruses. However, numerous obstacles prevent the effective control of MPXV, including social isolation and stigma, poor understanding of the disease dynamics, lack of adequate patient education, and public health strategies.

## Introduction and background

A rapidly spreading epidemic of monkeypox virus (MPXV), a once-common but neglected virus, has gained prominent attention in the midst of the COVID-19 pandemic. In May 2022, a cluster of pox-like diseases emerged in Europe, the United States, and the United Kingdom. The first MPXV outbreak was reported in Denmark in 1958 from a monkey in a research facility. In 1970, the first human case of MPXV was reported by a patient in the Democratic Republic of the Congo. The disease first spread outside Africa in May 2003, and subsequent cases in rats and dogs were linked to the importation of small animals from Ghana, West Africa [1]. According to the World Health Organization (WHO), the first index case of MPXV in the United Kingdom was identified on May 7, 2020, in a person with a travel history to Nigeria. Four new cases were identified that had no connection to previous cases or travel history but were men who were identified as having had sex with other men; the source of infection remains unknown. Consequently, the virus is spreading throughout the population [2].

**Figure 1:**
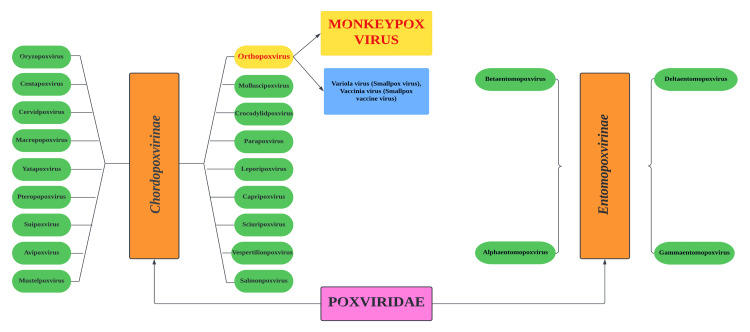
Poxviridae virus family Image credit: This image was created by Dr. Maleesha Jayasinghe, one of the authors of this study.

It is a brick-shaped, encapsulated, double-stranded DNA virus closely linked to the now-extinct variola virus, smallpox [3]. MPXV virus (MPXV) is a member of the *Poxviridae *family and the *Orthopoxvirus *genus (Figure 1).

The two distinct MPXV clades known as West African and Congo Basin are named for the regions in which they are found. Despite having varying degrees of severity, both of these clades result in a similar clinical condition [4].

The Congo Basin clade has been linked to death rates between 1% and 10%. In contrast to the Congo Basin clade, the West African clade is associated with a fatality rate of less than 3%. There are substantial variations in mortality rates, which are susceptible to case ascertainment bias. MPXV has been linked to severe adverse effects, including pneumonitis, vision-threatening keratitis, encephalitis, and subsequent bacterial infections. The MPXV from Central Africa is more likely to spread from person to person because it is more virulent than the West African clade. The geographic divide between the two virus clades is Cameroon, the only country where both virus clades have caused infections [5].

When the genomics of the Central African and West African strains was compared, it was discovered that the West African clade's open reading frames had deletions and fragmentations, contributing to its lower virulence than the Central African strain. In human cells derived from MPXV patients that were previously infected, Central African MPXV prevents T-cell receptor-mediated T-cell activation and the production of inflammatory cytokines [6]. Hammarlund et al. found that a low viral load of MPXV reduced T-cell-mediated cytokine responses by 80%, suggesting that MPXV may cause immunomodulation by inhibiting the response of host T-cells [7]. It has been suggested that the MPXV virus inhibitor of complement enzymes, which refers to a gene that inhibits complement enzymes and is absent in the West African strain, is a significant immune-modulating factor causing the increased virulence of Central African strains. In addition, the strain from Central Africa selectively inhibits the activity of host responses such as apoptosis. Studies of gene transcription have shown that Central African MPXV seems to selectively stop the transcription of genes involved in the host's immune system [6].

**Figure 2:**
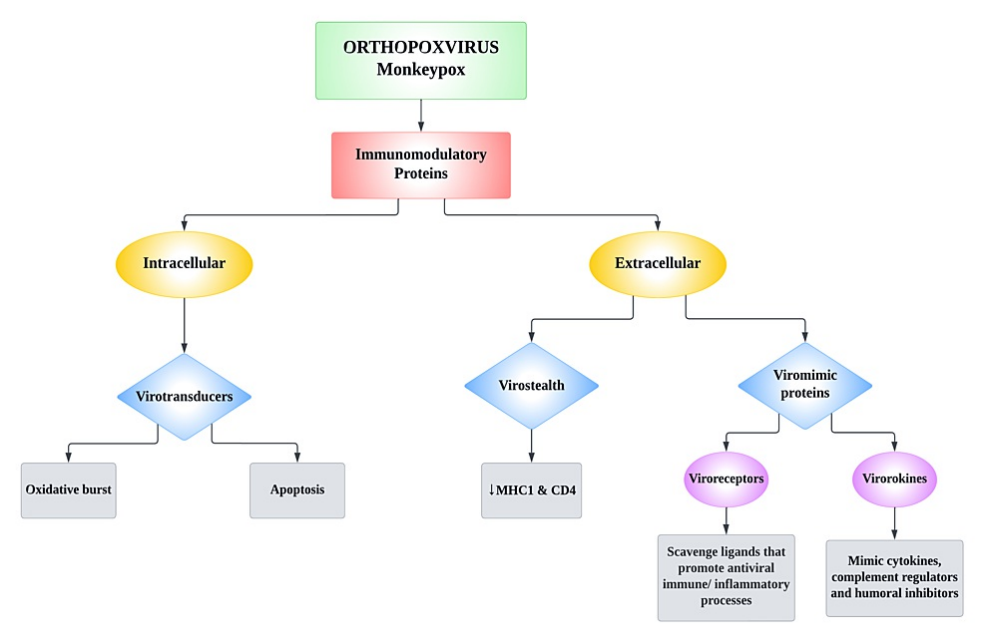
Effects of immunomodulatory proteins on host's immune system Image credit: This image was created by Dr. Maleesha Jayasinghe, one of the authors of this study. MHC: Major histocompatibility complex; CD4: Cluster of differentiation 4.

The larger size of poxviruses makes it more difficult for MPXV to traverse gap junctions and breach host defenses. It also hinders the virus' ability to replicate rapidly. Orthopoxviruses (OPXs) require a more coherent approach to survive inside the host. Because of the OPXs’ larger size, the immune system of the individual is notified very early on and can easily mount an immune response. In order to circumvent the host immune system, OPXs are equipped with molecules encoded by virulence genes that act as immunomodulators that target the host's immune system [8]. Figure 2 depicts the effects of immunomodulatory proteins on the host's immune system.

As a new threat to global health that can spread across borders and be transmitted further, monkeypox (MPX) has drawn the attention of researchers, epidemiologists, and medical professionals. Optimal infection control and treatment strategies for this potentially dangerous pathogen are not yet established. The disease can be managed through immunizations, good hygiene habits, and isolation or quarantine for the patients and their contacts.

It is worth taking note that the majority of the cases that have been reported have come from the men who have sex with men (MSM) population, who also face a higher risk of contracting HIV/AIDS and other sexually transmitted infections (STIs). This aspect of the current outbreak, in which the virus explicitly targets this population to spread, has not been documented. Clinical microbiologists are still unsure whether MPXV is transmitted through sexual contact. There have been reports of the MPXV spreading among HIV-positive individuals (PLHIV) who are receiving combination antiretroviral therapy (cART) and have entirely suppressed viremia in Spain [9]. Therefore, closely monitoring the pathogenic mechanisms of MPXV and HIV coinfection is necessary. It is unknown how the immune systems of people living with HIV (PLWH) will respond to MPXV because they have weakened immune systems. Other predisposing factors, such as coinfection with MPXV and other STIs, such as hepatitis B or C, will require close monitoring over the next few days.

Even among more affluent and educated population segments, the disease dynamics of MPXV remain poorly understood. Individuals have resisted disease testing and disregarded quarantines with impunity. In general, cough hygiene is inadequate. In developing countries, hand hygiene is also questionable. Improved public health strategies, including controlled animal model studies and others, to prevent virus transmission are urgently required. Due to their increased susceptibility to infection, pregnant women, children, and the elderly require special consideration. In addition, it is crucial to implement screening tools in healthcare settings and use emerging clinical case definitions to help identify cases and determine the scope of the outbreak. To prevent the spread of new infections and break the chain of transmission, it is essential to promptly isolate cases, closely monitor them, and vaccinate close contacts and medical personnel exposed to high-risk situations.

The purpose of this review is to discuss the epidemiology, clinical manifestations, diagnosis, treatment, and prevention of MPXV.

## Review

The epidemiology of the monkeypox virus

Although thought to be a rare disease, MPXV has been attracting a great deal of attention in the present since its discovery in 1958 among a group of captive monkeys transported from Africa to Denmark. Although it was first discovered in monkeys, the largest group of animal reservoirs was identified as rodents (squirrels and giant pouched rats). Humans and monkeys are both considered hosts of MPXV, but there is a lack of evidence to understand how the virus behaves in nature and the risk factors. Particularly in the past, MPXV has been geographically endemic to West Africa and Central Africa, including the Democratic Republic of Congo (DRC), Republic of the Congo, Cameroon, Central African Republic, Nigeria, Ivory Coast, Liberia, Sierra Leone, Gabon, and South Sudan, where intermittent attacks are reported. The reported number of cases has increased over the past 20 years, particularly in forested areas and among those populations who have not been vaccinated for the smallpox virus. In endemic regions, as the cross-protective immunity of the population decreases, non-vaccinated age groups have led to an increase in susceptibility [10].

Rather than singular case reports, there was a more significant increase in the total number of cases from 2000-2019 across a few African countries, including DRC, Cameroon, Liberia, and Nigeria. Until 2003, MPXV had not been detected outside the African continent, when 47 confirmed cases were found in the United States (US). According to studies done regarding this outbreak, the virus entered the US via a shipment made from Ghana, containing a few species of African rodents. A few cases were reported in Singapore, Israel, and the UK during the 2018-2019 time period among people with a travel history specifically to Nigeria. In the recent past, the DRC has been rated as the most affected country by MPXV, where 4594 suspected cases were reported from January to September 2020 [11].

West African Versus Central African Clades

WHO has separated the MPXV virus into two genetic clades, including the Central African (Congo Basin) clade and the West African clade, of which the Congo Basin clade is more virulent than the other. The West African clade is responsible for the outbreaks in the US (2003), Nigeria (2017), Sierra Leone, and the cases associated with the positive travel history in Israel, the UK, and Singapore in 2019, thereby contributing more toward human-to-human transmission [12,13]. In contrast to the Congo Basin clade, which has resulted in thousands of infections over the years and has a mortality rate of 11% in those who are unvaccinated, the West African clade has fewer documented cases, is less severe, and has resulted in no reported deaths [14].

The MPXV cases reported in DRC showed the lowest incidence was in 2001 (0.64 per 100,000), where an increase in the incidence was demonstrated from 2001 to 2012 from 0.64 to 3.11 per 100,000 people. The increased incidence persisted even after the removal of the Tshupa and Sankuru districts as active MPXV surveillance had been implemented in both districts to see whether the areas with active surveillance were the main factor for the increase in disease reporting [15]. However, during the outbreak in the US in 2003, 23% of the reported cases had gotten the smallpox vaccination in their childhood, suggesting that smallpox vaccination does not offer absolute protection against the MPXV [16].

Among the cases reported before the outbreak in 2017 in Nigeria, the majority of 83% of the cases were reported to be among children less than 10-years old with the rare secondary transmission, but cases reported in the 2017 outbreak predominantly affected young adult males with inter-human transmission [17].

Current Outbreak

In the current pandemic of MPXV, there have been about 14,000 reported cases in 78 different countries, with the majority of the initial cases occurring in Europe, primarily in the UK. The most impacted countries by MPXV are Spain, Germany, the US, the UK, and France. As of 23rd July 2022, 2,891 cases have been documented in the US, with the highest incidence in New York, followed by California. In the current outbreak, 99% of the cases were reported among men, with an average age of 41 years [18].

There were 18 cases reported from the Netherlands in people who had no history of traveling to endemic areas, and the infected people have been identified as MSM [19].

**Figure 3:**
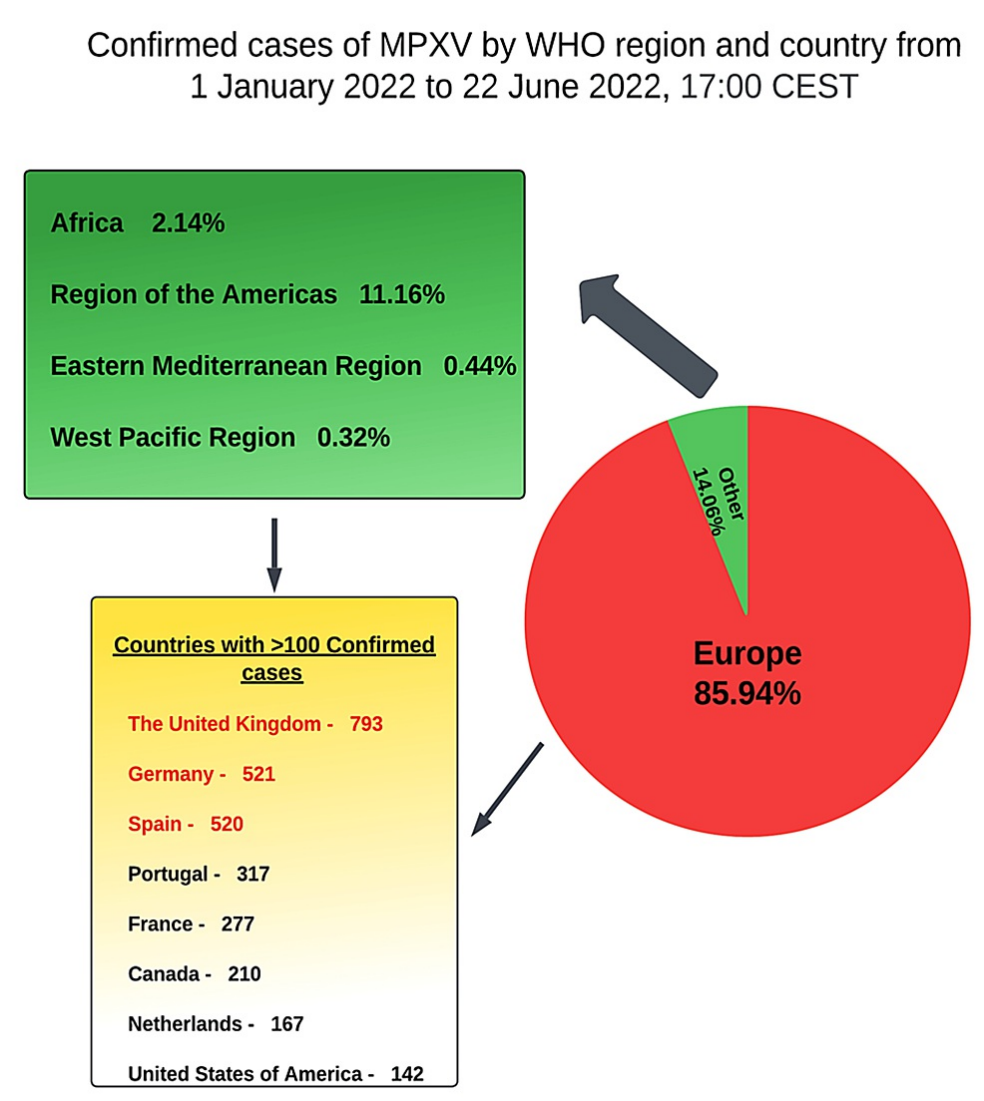
Confirmed cases of monkeypox virus in different countries Image credit: This image was created by Dr. Maleesha Jayasinghe, one of the authors of this study. MPXV: Monkeypox virus.

According to an active surveillance study done in the DRC, the risk of MPXV is inversely correlated with smallpox immunization. Those who received the smallpox vaccine before the end of the vaccination campaign in 1980 had a lower risk of getting infected by MPXV than the non-vaccinated people. The effectiveness of the vaccination is reduced in people born after 1980, likely due to the uncertain viability of the residual vaccine doses given at the time. The cross-protective immunity from previous smallpox vaccination is reported to be long-lasting against the genus Orthopoxvirus. Increased incidence, especially in immunocompromised hosts, may offer chances for the MPXV virus to mutate, potentially increasing its capacity for transmission, virulence, and pathogenicity. Furthermore, in a total of 528 cases identified between April and June 2022 from 16 different countries, 98% of the infected were homosexual or bisexual males, and 41% of the infected had a human immunodeficiency virus infection. In 95% of infected individuals, sexual transmission was thought to be the mode of transmission [20]. Figure 3 summarizes all the confirmed cases of MPXV in different countries from the 1st of January 2022 to the 22nd of June 2022.

Transmission of monkeypox

Since MPXVs are DNA viruses that are better at detecting and repairing gene mutations than RNA viruses, it is generally believed that they are unlikely to undergo sudden mutations that would significantly increase human-to-human transmission. O'Toole and Rambaut observed, however, that a cytidine deaminase known as the apolipoprotein B editing complex (APOBEC3) may be responsible for the recent rapid evolution of hMPXV1.4. By modifying DNA, cytosine deaminases generate double­-stranded breaks in switch regions. In human cells, activation-induced cytidine deaminase drives somatic mutations essential for antibody production. Retroviruses require APOBEC3 proteins, crucial in regulating single-strand DNA in viruses such as parvovirus, hepatitis B, and herpes [21].

According to another study, 41 out of 46 mutations were APOBEC3-like driving mutations. However, it is still unknown how a host antiviral gene contributes to the accumulation of MPXV mutations during an outbreak. Thus, monitoring the ongoing evolution of the MPXV's transmission capabilities is essential. At least 20 proteins were altered by the MPXV-2022 strains' present mutations. It is necessary to determine if these mutations play a part in the transmission or pathogenesis of the virus or if they help the virus evade the host's immune system. This study revealed that the 2022 outbreak strain contains nucleotide substitutions in OPG105 and OPG210. Both proteins have been altered in numerous MPXV lineages. Further analysis must assess how these two protein mutations affect viral function [22].

Clinical manifestations of monkeypox

MPXV can enter the body via the oropharynx, nasopharynx, and intradermal routes. It replicates at the site of inoculation and spreads to nearby lymph nodes. Viremia causes viral propagation and the seeding of other organs [23].

The incubation time ranges from five to 21 days, typically between six to 13 days. The sickness begins with a vague prodromal phase lasting between zero to five days. Some clinical symptoms include fever, chills, headache, fatigue, myalgia, lymph node swelling, and back pain. Lymphadenopathy, which may be unilateral/bilateral cervical, axillary, or inguinal, will occur with the onset of fever [24]. Another uncommon and hard-to-diagnose clinical manifestation is dysphagia, which may be the first sign of the disease [25]. Neurological and psychiatric symptoms such as confusion, encephalitis, strokes, and seizures can also occur [26]. Painful rashes of varying sizes are a hallmark of MPXV, appearing anywhere from one to five days after the onset of fever. Depending on the case, it could last anywhere from two to four weeks. Rash symptoms typically begin as macules on the face and spread to the rest of the body, including the hands and feet. Macular, papular, vesicular, and pustular stages characterize the rash's progression. The face (98%), palms and soles (95%), oral mucous membrane (70%), genitalia (28%), and conjunctiva (20%) are the most prevalent sites of infection. On day 3, the lesions transform into papules; on day 4, they become vesicles (raised and fluid-filled); on day 6 or 7, they become pustules, prominently elevated, filled with opaque fluid, rigid, and deep-seated. They may umbilicate or merge into one another. They dry out by the second week's end and form crusts [24].

A descriptive case series conducted in 2022 showed that rectal pain (due to rectal perforation) and penile edema could be identified in MPXV. One hundred and ninety-seven patients participated in the case series, and 196 identified themselves as gay or bisexual. Among these people, 71 showed rectal pain, and 31 showed penile edema [27].

Symptoms usually resolve in most people within two to four weeks. Nevertheless, immunocompromised patients, younger children, pregnant patients, and those absent from previous smallpox vaccination can get severe complications from the disease, such as bronchopneumonia, sepsis, encephalitis, sight-threatening keratitis, and secondary bacterial infections [24].

The smallpox vaccine was thought to be the most effective method for combating MPXV. Comparing the protein composition and identifying sequence identities between the vaccine strain (MVA-BN) and the MPXV-2022 consensus sequence revealed that the vaccine strain differed by only 10 proteins from the MPXV vaccine strain (MVA-BN) [28]. Due to their similarities, the viral symptom profiles of smallpox and MPXV are identical; consequently, it is necessary to differentiate these two viruses to manage both diseases effectively. Nevertheless, since smallpox has been abolished, the most pressing difficulty in contemporary clinical diagnosis is distinguishing between human MPX and chickenpox. Since many MPX cases are unrecognized, their incidence and geographic distribution remain uncertain. In 1981, special surveillance in three regions of Zaire revealed a substantial increase in MPXV cases. Errors in diagnosis have raised the possibility that some cases of MPXV were misdiagnosed as other eruptive diseases. This study delivers an in-depth analysis of the causes of these diagnostic errors in areas where health professionals and the general public are aware of human MPXV. Laboratory testing was conducted on 977 individuals with skin eruptions in Zaire between 1981 and 1986 who were not clinically diagnosed with MPXV; 3.3% of MPXV cases were detected in 730 patients diagnosed with chickenpox, 7.3% of MPXV cases were noted in patients diagnosed with "atypical chickenpox," and 6.1% of MPXV cases were found in patients with a skin rash for whom a clinical diagnosis could not be determined. Regional pleomorphism, uncertain body distribution of skin eruptions, and centripetal distribution of skin lesions were primarily responsible for the diagnostic challenges. In 76% of misdiagnosed patients, lymph node enlargement was found. The biggest diagnostic problem in the absence of smallpox is identifying human MPXV from chickenpox. The presence of lymphadenopathy, pre-eruptive fever, and delayed progression of skin lesions are the most important clinical markers for the correct diagnosis of MPXV [29].

Patients have been shown to be co-infected with MPX and varicella zoster virus (VZV) in studies. Hughes et al. collected samples from multiple skin lesions from individual patients [27]. They investigated the causal association of these coinfections through cell culture and electron microscopy analysis of dual positive original samples. The evaluation of samples collected from cases of coinfection revealed that MPX and VZV cause distinctive lesions. Even though only a limited number of lesion samples have been examined, neither OPX nor VZV virions have been detected in a single lesion sample by electron microscopy. However, there were differences in the severity and reported symptoms between those with coinfections and those with MPX or VZV alone. Compared to cases with VZV alone, coinfection cases were more likely to report MPX illness-related signs and symptoms and had a higher lesion burden. However, cases with coinfection were less likely to report these symptoms and had a substantially lower rash burden than MPX-only cases. These findings suggest that coinfection with these two rash viruses may mitigate the overall severity of infection. For the simultaneous detection of both viruses from a single patient, co-circulation of both viruses or an unidentified pathogenic factor specific to infection with one or both viruses has been noted as an explanation. However, the mechanism underlying MPX and VZV coinfection remains unknown. One hypothesis says that initial infection with either virus makes the patient's immune system more susceptible to a secondary infection. Another possibility is that MPXV infection directly activates VZV, resulting in herpes zoster [30]. Table 1 summarizes the distinguishing characteristics of MPXV, variola virus (smallpox), and VZV.

**Table 1:** Distinguishing features of monkeypox virus, smallpox virus, and varicella zoster virus Credit: This table was created by Dr. Maleesha Jayasinghe, one of the authors of this study. MPXV: Monkeypox virus.

	Smallpox	MPXV	Varicella zoster
Family	Poxviridae, orthopoxvirus	Poxviridae, orthopoxvirus	Herpesviridae
Species	Variola	MPXV	Human alphaherpesvirus 3
Reservoir	Only humans	Zoonotic	Only humans
Clades/strains	Variola major and Variola minor	Central African and West African	Clades 1 and 3 - European/North American strains; Clade 2 - Asian strains (especially Japan); Clade 5 - India; Clade 4 - Europe (geographic origins need further documentation)
Reproduction rate (number)	3.5-6	1-2.5	3.7 and 5.0
Clinical features	2–4 days before the rash. The rash starts as small red spots on the tongue and mouth. Then it appears on the skin, starting on the face and spreading to arms and legs, and then palms and soles. The rash eventually forms a scab that falls off. Lymph nodes are absent.	1–5 days before the rash. The rash often starts on the face and then spreads to other parts of the body, including the palms and soles. The rash eventually forms a scab that falls off. Lymph nodes are present.	1–2 days before the rash. Rashes are itchy; blister-like rash starts first on the chest, back, and face, and then spreads over the entire body. Absent on palms and soles. Lymph nodes are absent.
Time between catching it and the symptoms	7–19 days	5–21 days	10–21 days
Duration of illness	Up to 30% of cases (depending on the type)	1%–10% of cases (depending on strain)	Rare
Fatality rate	Up to 30% (depending on strain)	1%–10% (depending on strain)	Rare
Vaccine	Smallpox vaccine	Smallpox vaccine	Chickenpox vaccine

Diagnosis of monkeypox virus

In order to diagnose MPX, medical professionals must collect an appropriate specimen and send it to a laboratory for confirmation of the virus. MPXV can be identified using various diagnostic methods, including genetic, phenotypic, immunological, and electron microscopy. Diagnosing MPXV based on clinical symptoms (phenotypic method) is necessary to identify potential cases during examination [31].

Due to its high accuracy and sensitivity, polymerase chain reaction (PCR) is the standard test for detecting MPX-specific DNA sequences. The recommended diagnostic samples for genetic testing are obtained from skin lesions (from the surface of the lesion, exudate, or crust) or, when possible, a biopsy. However, sample contamination is a possibility in highly sensitive examinations. In addition, these tests require expensive equipment, reagents, and expert techniques, which restricts their widespread use in clinical practice [30].

Noe et al. outlined the first two MPX cases in Germany. In this study, patient samples were analyzed using a quantitative polymerase chain reaction (qPCR) assay for the simultaneous detection of *orthopoxvirus *species, such as the RealStar Orthopoxvirus kit (Altona Diagnostics, Germany). Positive samples were further examined with an MPXV-specific qPCR. Throat and skin swab samples had the highest average number of genome copies per milliliter, while ethylenediaminetetraacetic acid (EDTA) in blood and sperm also contained MPXV genome copies. This was the first study in which MPXV DNA was detected in sperm samples [32].

In a separate study, Li et al. reported the development of three new real-time PCR assays based on TaqMan probe technology: the MPXV West African-specific, Congo Basin strain-specific, and MPXV generic assays. In the validation study with multiple platforms and various PCR reagent kits, these new assays demonstrated reasonable specificity and sensitivity, improving the rapid detection and differentiation of MPXV infections [33].

Clinically, the skin rash resembles the common and modified forms of smallpox as well as classical and atypical chickenpox. In addition, the clinical course of MPXV in smallpox-vaccinated versus unvaccinated patients is significantly different. Thus, it is essential to recognize key histological characteristics of the disease and distinguish them from those caused by other viruses. Clinical progression of MPXV lesions is mirrored histologically by ballooning degeneration of basal keratinocytes and spongiosis of a mildly acanthotic epidermis, followed by full-thickness necrosis of a severely acanthotic epidermis containing few viable keratinocytes. There is a lichenoid-mixed inflammatory cell infiltrate, which exhibits progressive exocytosis in conjunction with keratinocyte necrosis. There is also inflammation of the superficial and deep vascular plexus, eccrine units, and follicles. Multinucleated syncytial keratinocytes exhibit the cytopathic effect of the virus. The viral antigen is detected immunohistochemically within keratinocytes of the lesional epidermis, follicular and eccrine epithelium, and a small number of dermal mononuclear cells. The electron microscopy of keratinocyte cytoplasm reveals virions at various stages of assembly [34]. Even though immunological methods permit sensitive detection of IgG or IgM antibodies against MPX using the enzyme-linked immunosorbent assay (ELISA) test, it cannot be considered a qualitative test for detecting human MPXV. Electron microscopy can differentiate between OPX and herpes simplex viruses. It suggests that MPXV is a member of the *poxviridae *family. Nonetheless, OPXs are indistinguishable from one another, necessitating more specific testing to diagnose [31].

GeneXpert also has a place in the diagnosis of MPXV. Recent studies indicate that the GeneXpert MPX/OPX assay will be the solution to many delayed MPX diagnoses. GeneXpert MPX/OPX does not require complex laboratory environments, such as real-time PCR, and can be utilized in rural field settings. It uses a specialized cartridge to prepare samples. Diagnostic specimen samples consist of crusts and vesicular swabs. Additionally, it can detect MPX infection at any stage of the rash, enabling quicker disease management. The MPXV detection assay, GeneXpert MPX/OPX, is highly sensitive and specific. There are many benefits to cartridge preparation requiring only a small sample, such as lowering the risk associated with needles, reducing the risk of contamination, and increasing the testing officer's safety. GeneXpert cartridges will be widely utilized because they are inexpensive [35].

Management of monkeypox

There is no proven cure for MPXV yet. The majority of MPXV infection patients recover without the need for any medical treatment. To reduce gastrointestinal fluid loss, certain patients with gastrointestinal symptoms will require oral or intravenous rehydration [36].

Antivirals

Based on animal studies, many antivirals, such as tecovirimat, brincidofovir, and cidofovir, that were initially approved for treating smallpox have been playing a role in managing MPXV infections. In a comprehensive safety study of 359 human volunteers who were given tecovirimat, similar side effects were observed between placebo and tecovirimat. A combination of tecovirimat and brincidofovir may be used to treat severely diseased patients. Small studies have shown that dual therapy with tecovirimat and vaccinia immune globulin (VIG) is beneficial in patients having complications from the smallpox vaccine, such as eczema vaccinatum and progressive vaccinia. The US Strategic National Stockpile (SNS) stores tecovirimat in oral capsules and intravenous vials. Studies have shown that the clinically beneficial dose of tecovirimat for adults is 600 mg twice daily for 14 days; for children (13 kg to less than 25 kg), it is 200 mg BID for 14 days; for those (25 kg to less than 40 kg), it is 400 mg twice daily for 14 days; and for those (40 kg or more), it is 600 mg twice daily for 14 days. The common side effects of tecovirimat are headaches, nausea, abdominal pain, vomiting, and injection-site reactions. Tecovirimat has no major contraindications except that intravenous injections are not recommended in patients with severe renal impairment. The first oral antiviral drug approved by the US FDA in 2018 for treating smallpox is tecovirimat (commonly known as Tpoxx) [35]. Although the dosage for these drugs has been studied in humans, their efficacy has not been entirely determined [37].

Following its approval in May 2022 for the treatment of MPXV, it is now the preferred option [38]. Cell cultures containing multiple isolates of the variola virus were found to be inhibited by tecovirimat. Tecovirimat works by blocking the final steps in viral maturation by inhibiting the viral envelope protein VP37, thus preventing the release from the infected cell and the spread of the virus within an infected host. In many lethal animal studies, tecovirimat has shown excellent oral bioavailability while exhibiting adequate broad-spectrum protection against all OPXs that are known to infect humans [39].

The US FDA approved brincidofovir (also known as Tembexa) in June 2021 to treat smallpox. Brincidofovir (oral) is a prodrug of the intravenous drug "cidofovir" (also known as Vistide) and may appear safer than cidofovir with less renal toxicity [36,40]. The Centers for Disease Control and Prevention (CDC) has provided guidance on using cidofovir to treat life-threatening MPXV and not for prophylaxis [38]. Both brincidofovir and cidofovir block OPX DNA polymerase-mediated viral DNA synthesis in a manner that is analogous to one another. Clinical statistics on the efficacy of cidofovir against MPXV in humans are insufficient, although positive results have been seen in animal studies. The recommended dose of brincidofovir for adults weighing 48 kg is 200 mg once weekly for two doses of oral tablets; for adults and pediatric patients weighing 10 kg to less than 48 kg, the dose is 4 mg/kg of the oral suspension once weekly for two doses. For children weighing 10 kg, the dose is 6 mg/kg of the oral suspension once weekly for two doses. The dosage of cidofovir is 5 mg/kg once a week for two weeks, followed by a 5 mg/kg IV once every other week. Compared to cidofovir, brincidofovir is associated with diarrhea, nausea, vomiting, and abdominal discomfort. Cidofovir is associated with reduced serum bicarbonate, proteinuria, neutropenia, infection, eye hypotonia, iritis, uveitis, nephrotoxicity, and fever. Brincidofovir is not recommended for pregnant and breastfeeding women. In contrast, cidofovir is not given to people with hypersensitivity reactions to cidofovir or any of its components and in patients with severe renal impairment [36].

Vaccinia Immune Globulin

VIG is a hyperimmune globulin preparation rich in antibodies that provide passive immunity against specific diseases. VIG is used intravenously and is FDA-approved for treating specific complications of vaccinia vaccination, including severe generalized vaccinia, eczema vaccinatum, progressive vaccinia, ocular vaccinia, post-vaccine central nervous system complications, vaccinia infections in individuals with skin conditions, and atypical infections caused by vaccinia virus [35,38]. While VIG is a potential treatment for MPXV and smallpox, statistics on its efficacy are largely insufficient. VIG has not been used for MPXV or smallpox in human trials. Vaccination against vaccinia is not advocated for patients with severe T-cell immunodeficiency unless there is a history of exposure; alternatively, VIG may be administered. Treatment with VIG should be administered under an IND application. The dose of intravenous immunoglobulin (IVIG) to be given as soon as symptoms manifest is 6000 U/kg. If the symptoms become severe, the IVIG may be repeated depending upon the response to treatment; if the patient does not respond to the initial dose, 9000 U/kg may be considered. The common side effects of IVIG are headaches, nausea, rigors, and dizziness. IVIG is contraindicated in people with a history of isolated vaccinia keratitis, anaphylactic or severe systemic reactions to human globins, Ig A deficiency with antibodies against Ig A, and Ig A hypersensitivity [36].

Whenever possible, treatment for MPXV infection should ideally be administered within a clinical trial to generate long-term data that could inform future decisions regarding how to best treat patients. Clinicians are encouraged to coordinate treatment plans and strategies with infectious disease specialists and public health officials.

Prevention of Monkeypox: Vaccination

Statistics indicate that prior smallpox vaccination may protect against the MPXV and reduce its clinical manifestations. JYNNEOS™ (also known as IMVAMUNE, IMVANEX, MVA-BN), ACAM2000®, and the Aventis Pasteur Smallpox Vaccine (APSV) are the three smallpox vaccines in the US SNS. JYNNEOS™ and ACAM2000® are currently licensed to treat smallpox, whereas the use of APSV for smallpox is permitted under an investigational new drug (IND) protocol. JYNNEOS™ is produced using the modified vaccinia Ankara-Bavarian Nordic (MVA-BN strain) and is a live, attenuated, non-replicating OPX. It was granted approval by the FDA in September 2019 [35]. Previous statistics have shown that smallpox immunization with the vaccinia virus was about 85% effective against MPXV [40].

ACAM2000® also contains the live form of the vaccinia virus. However, it is replication-competent in contrast to JYNNEOS™. It was certified by the FDA in August 2007, replacing the previously available OPX vaccine Dryvax®, which was removed from the market by the manufacturer. The CDC authorizes the use of ACAM2000® under an emergency access IND protocol during an outbreak of MPXV. The FDA evaluated the efficacy of JYNNEOS™ and ACAM2000® by comparing the immunologic response of JYNNEOS™ to that of ACAM2000® and integrated supportive animal studies. APSV is a replication-competent vaccinia vaccine with a safety profile anticipated to be similar to ACAM2000® that may be used as a prophylaxis for smallpox under an IND or Emergency Use Authorization (EUA) when the licensed vaccines are unavailable or contraindicated. However, whether this vaccine could be used for MPX is still unknown. [36]. Table 2 shows FDA-approved smallpox vaccines that can be used for MPXV.

**Table 2:** Food and Drug Administration-approved smallpox vaccines Credit: This table was created by Avinash Sewsurn, one of the authors of this study. APSV: Aventis Pasteur Smallpox Vaccine; IND: Investigational new drug; EUA: Emergency Use Authorization; MPXV: Monkeypox virus.

Smallpox Vaccines	ACAM2000^®^	JYNNEOS^™^	APSV
Manufacturer	Emergent BioSolutions (previously Sanofi Pasteur Biologics Co.)	Bavarian Nordic	Emergent BioSolutions (previously Sanofi Pasteur Biologics Co.)
Type of vaccine	Replicating, live vaccinia virus	Live, attenuated, non-replicating virus	Replication-competent vaccinia vaccine
Method of administration	Percutaneously as a single dose using a bifurcated needle by the multiple puncture technique	Two subcutaneous doses, with the second dose administered 28 days later	Multiple puncture technique using a bifurcated needle to administer a single dose percutaneously
Indication	Provides active immunity against smallpox disease to individuals at high risk of infection	They are administered to adults who are 18 years and older and at high risk for contracting smallpox or MPXV.	IND or EUA
Adverse effects	Risk of accidental inoculation and autoinoculation, vertical transmission can occur that can be fatal to the fetus, uncontrolled viral replication in some individuals (particularly the immunocompromised), eczema vaccinatum in individuals with atopic dermatitis or eczema, inoculation site reactions, myopericarditis, and post-vaccine encephalitis	Injection site reactions (pain, redness, swelling, a hard lump, and itching), muscle pain, headache, and fatigue	Risk of accidental inoculation and autoinoculation, vertical transmission can occur that can be fatal to the fetus, uncontrolled viral replication in some individuals (particularly immunocompromised), eczema vaccinatum in individuals with atopic dermatitis or eczema, and significant cutaneous reaction at the site of inoculation can be observed.

Pre-exposure Prophylaxis (PrEP)

The Advisory Committee and Immunization Practices (ACIP) advocates vaccination for people at risk, especially those exposed at their occupational places to OPXs, such as laboratory staff performing diagnostic testing for OPXs and assigned response teams. In addition, healthcare workers who administer ACAM2000® or care for patients infected with reproducing OPXs can be immunized based on shared clinical decision-making. ACIP contraindications for primary vaccinees and re-vaccinees of ACAM2000® for PrEP include a history or presence of atopic dermatitis or eczema, other active exfoliative skin conditions, immunosuppression conditions (e.g., HIV-infected persons), pregnancy, age < one year, breastfeeding, severe vaccine component allergy, known underlying heart disease (e.g., coronary artery disease or cardiomyopathy), three or more known major cardiac risk factors (in primary vaccinees only) in high-risk populations with undetected HIV, and a population from which MPXV may have a wider spread (e.g., sex workers). It is also not given to laboratory and healthcare personnel whose close contacts (household contacts and sexual contacts) have similar contraindications as those listed above. ACIP contraindications for JYNNEOS™ vaccinees for PrEP are the presence of only a severe vaccine component allergy [36].

Post-exposure Prophylaxis (PEP)

Close interaction with infected individuals for long periods of time increases the likelihood of MPXV transmission. Short-duration interactions and those performed using appropriate personal protective equipment per standard precautions are not at high risk and typically do not require PEP. The CDC recommends the prophylactic administration of the first dose of the vaccine within four days of exposure. Vaccination may lower the disease symptoms if given four to 14 days after exposure. However, this may not prevent the disease's onset [35]. Table 3 shows the high, intermediate, and low-risk exposures to MPXV infection.

**Table 3:** High, intermediate, and low-risk exposures to monkeypox virus infection Credit: This table was created by Avinash Sewsurn, one of the authors of this study. PEP: Post-exposure prophylaxis.

Risk exposure	High	Intermediate	Low
Causes	Unprotected and prolonged contact between a person's skin and the mucous membranes, skin, lesions, bodily fluids (e.g., any sexual contact, accidental splashes of patient saliva to the eyes or oral cavity of a person, not wearing gloves while handling a patient), or contaminated clothing or towels, being in the patient's room or within six feet of a patient during any procedures exposed to aerosols from oral secretions, skin lesions, not wearing an N95 or equivalent respirator (or higher), and eye protection	Being within six feet for three hours or more of a patient while not wearing a mask, coming into contact between sleeves and other parts of an individual's clothing as well as the patient's skin lesions or bodily fluids or their soiled linens or dressings (e.g., turning, bathing, or assisting with transfer) while wearing gloves but not wearing a gown	Low or uncertain exposures
Management	Monitor and give PEP vaccination	Monitor and give PEP vaccination if the benefits outweigh the risks	Monitor, may not need PEP vaccination

## Conclusions

MPX can present with fever, chills, headache, fatigue, and myalgia. Lymphadenopathy, pre-eruptive fever, and painful skin lesions serve as more specific clinical markers. Most patients will recover with no or minimal medical intervention. Though optimal infection control and treatment strategies are not yet well established, the current focus is to manage the disease through immunizations, good hygiene habits, and isolation or quarantine for the patients and their contacts.
